# Mitochondrial Dynamics: Molecular Mechanisms, Related Primary Mitochondrial Disorders and Therapeutic Approaches

**DOI:** 10.3390/genes12020247

**Published:** 2021-02-10

**Authors:** Michela Di Nottia, Daniela Verrigni, Alessandra Torraco, Teresa Rizza, Enrico Bertini, Rosalba Carrozzo

**Affiliations:** Laboratory of Molecular Medicine, Unit of Muscular and Neurodegenerative Disorders, Bambino Gesù Children’s Hospital, IRCCS, 00146 Rome, Italy; michela.dinottia@opbg.net (M.D.N.); daniela.verrigni@opbg.net (D.V.); alessandra.torraco@opbg.net (A.T.); teresa.rizza@opbg.net (T.R.); enricosilvio.bertini@opbg.net (E.B.)

**Keywords:** mitochondrial dynamics, mitochondrial diseases, therapeutic approaches

## Abstract

Mitochondria do not exist as individual entities in the cell—conversely, they constitute an interconnected community governed by the constant and opposite process of fission and fusion. The mitochondrial fission leads to the formation of smaller mitochondria, promoting the biogenesis of new organelles. On the other hand, following the fusion process, mitochondria appear as longer and interconnected tubules, which enhance the communication with other organelles. Both fission and fusion are carried out by a small number of highly conserved guanosine triphosphatase proteins and their interactors. Disruption of this equilibrium has been associated with several pathological conditions, ranging from cancer to neurodegeneration, and mutations in genes involved in mitochondrial fission and fusion have been reported to be the cause of a subset of neurogenetic disorders.

## 1. Introduction

Mitochondria are a dynamic network of fusing and budding organelles, which are present in all eukaryotic cells, except for erythrocytes. They are characterized by a double membrane structure: The mitochondrial outer membrane (MOM), rich in porins that allow passage of small molecules, and the mitochondrial inner membrane (IMM), which forms many invaginations (named cristae) and is impermeable to most solutes. Mitochondria are considered the “powerhouse” of the cell, since they are responsible for producing the majority of the cellular energy in the form of adenosine triphosphate (ATP). Beyond their primary role as ATP generators, mitochondria are also involved in apoptosis, heme and steroid synthesis, Ca2+ and metabolic regulation, cell-cycle progression, as well as in signaling pathways related to the immunological process [1]. These organelles can be considered semi-autonomous, since they house their own genome, the mitochondrial DNA (mtDNA), as well as the RNA and the protein-synthesizing apparatus, which are necessary for the functioning of the oxidative phosphorylation (OXPHOS) that is the process through which ATP is produced. The OXPHOS system is composed of five multi-subunit enzyme complexes (I–V), whose concerted action is required for ATP synthesis [2]. About 92 structural proteins are requested to build the OXPHOS system, of which only 13 are encoded by the mtDNA, while all the remaining are derived from nuclear genes and then imported into mitochondria [3]. Additional 1500 proteins encoded by the nuclear DNA are destined for the mitochondria, some of them are crucial for the OXPHOS system assembly, and others take part in several processes targeted for mitochondrial homeostasis, such as regulating mtDNA replication, transcription, and translation, preserving the redox homeostasis, and eliminating damaged mitochondria from the cell and the maintenance of the mitochondrial dynamics [4]. Among all, mitochondrial dynamics is the major player in safeguarding “mitochondrial welfare”. In fact, the ability of mitochondria to fuse, divide, and change their morphology, allowing them to carry out critical functions, such as local energy supply, inter-organelle cross-talk, mixing of mitochondrial content, mtDNA maintenance, regulation of mitochondria distribution during mitosis, and segregation of damaged mitochondria [5].

Mutations in any of the genes involved in mitochondrial functions could be the cause of mitochondrial diseases (MDs), a heterogeneous group of disorders, in which the pathogenic variants primarily or secondarily can affect the OXPHOS activity, the organelle structure and dynamics, or other mitochondrial process—such as the production of cofactors and vitamins, fatty acid oxidation, and Krebs cycle [6]. MDs are the most common group of neurometabolic disorders with a prevalence of 1:5000 [7] and are characterized by high clinical heterogeneity. In fact, they can “present with any symptom in any organ at any age” [8]. Besides the neuromuscular system, many organs, with a high energy demand, can exhibit symptoms and signs, such as the heart, eyes, ears, kidneys, endocrine glands, liver, bone marrow, and gastrointestinal tract [9].

## 2. Overview of Mitochondrial Dynamics

**Figure 1:**
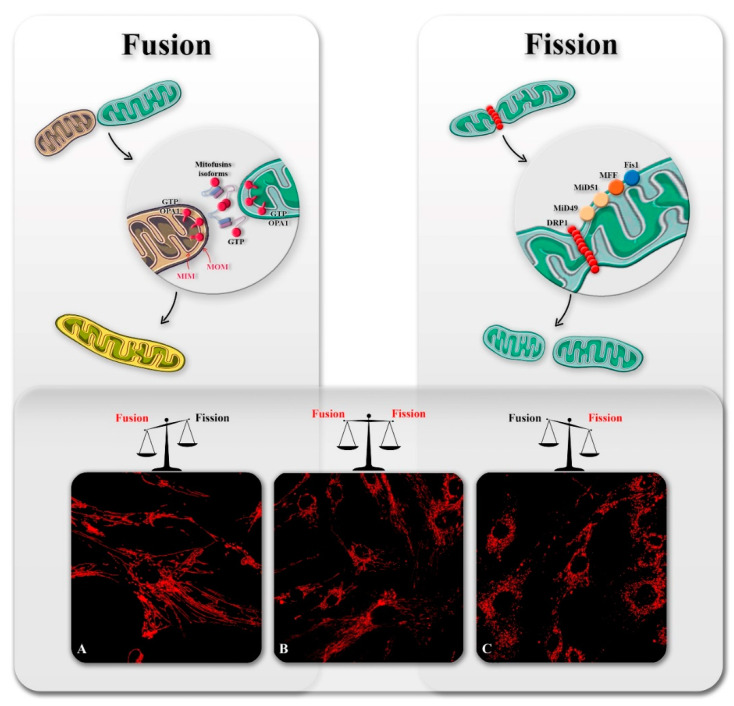
Mechanism and regulation of mitochondrial dynamics. Mitochondrial fusion (upper left panel) is mediated by large dynamin-related GTPase proteins Mfn1, Mfn2, and OPA1. Mitochondrial outer membrane (MOM) fusion is mediated by Mfn1 and Mfn2, whereas mitochondrial inner membrane (MIM) fusion is mediated by OPA1. Mitochondria fission (upper right panel) requires the recruitment of Drp1 from cytosol to mitochondria. Drp1 binds to four Drp1 receptor proteins Fis1, Mff, MID49, and MID51. Below representative images of hyperfused (**A**), regular (**B**), and fragmented (**C**) fibroblast mitochondrial network.

Mitochondria do not exist as individual entities in the cell; they indeed constitute an interconnected community governed by the constant and opposite process of fission and fusion, which dictate the organelle shape, size, and positioning to meet the metabolic needs of the cell [10]. The mitochondrial fission leads to the formation of smaller mitochondria, promoting the biogenesis of new organelles, especially during cell division, their transport, and redistribution and facilitating the segregation of damaged mitochondria. On the other hand, following the fusion process, mitochondria appear as longer and interconnected tubules, which enhance the communication with other organelles and acquire the ability to mix their content, diluting the accumulated mitochondrial DNA mutations and oxidized proteins (Figure 1) [4].

Both fission and fusion are carried out by a small number of highly conserved guanosine triphosphatases (GTPases) proteins and their interactors, whose tuned and regulated actions are required to balance these opposite processes. Disruption of this equilibrium affects mitochondrial homeostasis, and not surprisingly, it has been associated with several pathological conditions, ranging from cancer to neurodegeneration, and mutations in genes involved in mitochondrial fission and fusion have been reported to be the cause of a subset of neurodegenerative disorders [11,12].

### 2.1. Mitochondrial Fission

The result of the mitochondrial fission process is the division of a mitochondrion into two smaller organelles. The main executor of this reaction is a GTP-hydrolyzing enzyme, the dynamin-related protein 1 (Drp1). Drp1 is a member of the dynamin superfamily GTPases, and similarly to other dynamins, through oligomerization and conformational changes, it acts mainly in membrane remodeling. The primary sequence of this protein consists of four conserved regions: The N-terminal GTPase domain, the middle, the variable, and the GTPase effector domain (GED) in C-terminal. Drp1 is mostly localized in the cytosol as a mixture of dimers and tetramers, and in response to specific cellular signals, it is recruited via receptors to the MOM, where it oligomerizes into filaments that wrap up and narrow the membranes. Furthermore, the GTP hydrolysis triggers conformational changes in Drp1 oligomers that generate the mechanical force to promote mitochondrial membrane scission [13,14,15]. Unlike other dynamins, Drp1 does not contain a phospholipid-binding pleckstrin homology (PH) domain, which means that it slowly binds to membrane phospholipids, and its recruitment to the mitochondrial surface requires adaptor proteins [14]. In mammals, four proteins have been described to be involved in Drp1 recruitment to the OMM: Mitochondria fission factor (Mff), mitochondrial dynamics protein of 49 kDa (MID49), 51 kDa (MID51), and fission 1 (Fis1) [16].

Mff is a tail-anchored membrane protein and is considered the primary Drp1 receptor at both mitochondria and peroxisomes [17,18]. Mff is distributed in discrete foci along the OMM, where it specifically recruits high-oligomeric forms of Drp1. Moreover, it has been suggested that this protein increases Drp1 GTPase activity, thus promoting the Drp1 helices pinching around the mitochondrial membranes and consequently the division of mitochondria [19].

MIDs proteins, anchored to the OMM through an N-terminal transmembrane domain, are Drp1 receptors present on mitochondria and not on peroxisomes [20]. Crystal structures studies revealed that both MID49 and MID51 possess a noncanonical nucleotidyltransferase (NTase) domain, but they lack the critical residues required for transferase activity; however, it is possible that the fold of Ntase acts as a protein recruitment platform to assemble Drp1 oligomers on the OMM [21,22]. Anyway, structural differences have been reported between these two Drp1 receptors, for example, unlike MID49, MID51 does bind adenosine diphosphate (ADP) as a cofactor; moreover, MID49 assumes a monomer structure, whereas MID51 is dimeric. These differences may reflect their differential regulation of mitochondrial fission. To this purpose, it has been proposed that the dimeric structure of MID51 favors the recruitment of Drp1 at the basal state, which is believed to be dimeric. In addition, it has been suggested that the capability of MID51 to bind ADP enables it to sense the metabolic changes in the cell [21].

Fis1 was the first identified receptor for Drp1 in yeast [23,24], where it recruits Dnm1p (the yeast Drp1 homolog) through interaction with adaptor proteins Mdv1p and Caf4p [25]. However, the absence of Mdv1 and Caf4 homologs in mammals makes the role of the human Fis1 in the mitochondrial fission process ambiguous. In fact, it has been reported that Fis1 binds Drp1 and takes part in the Drp1-dependent mitochondrial fission [26,27,28]. Later, after the identification of MIDs and Mff receptors, the Fis1 role in mitochondrial fission has been reconsidered, and it has been demonstrated that Drp1 activity is not or not much influenced by silencing or overexpression of human Fis1 [16,17,29]. However, Fis1 has been reported to play a specific role in the mitophagy process, forming a complex with several ER proteins and Mff-recruited Drp1, thus promoting the mitochondrial fission required for mitochondrial autophagy [30]. The involvement of Fis1 in mitophagy is also supported by the demonstration that its overexpression triggers mitophagy, while Fis1 knockout severely reduces the mitochondrial removal rate [30,31,32]. In a recent work, it has been shown that Fis1 weakly binds Drp1, while it interacts robustly with Mfn1, Mfn2, and OPA1, inhibiting the GTPase activity of these proteins, which are necessary for the fusion process. These data reveal a novel function for Fis1 in regulating mitochondrial dynamics in mammals, according to which it promotes fission by acting as a negative regulator of the pro-fusion machinery [33].

Although Drp1 and its receptors/adaptors represent the core of the fission machinery, both the initial and the final steps of this process rely on the action of other cellular players, such as the endoplasmic reticulum (ER), the actin cytoskeleton, and another dynamin GTPase, dynamin-2 (Dnm2). It has been observed that mitochondrial fission occurs almost exclusively at the regions of ER-mitochondria-cytoskeleton contact [34], where ER membrane tubules enwrap the elongated mitochondria to mark sites of the imminent mitochondrial division [35]. Moreover, both myosin II and the ER-localized formin 2 protein have been suggested to drive the actin polymerization at the ER-mitochondrial interface, allowing efficient fission [36,37]. Given that the mitochondrial tubule has a diameter approximately fivefold larger than that of Drp1 helices, the ER-cytoskeletal machinery cooperation is required to pre-constrict mitochondria to a diameter that is permissive for DRP1 oligomer assembly [34]. While the role of Drp1 in membrane constriction is widely documented, its role in membrane scission is debated. In vitro studies have shown that Drp1 is not able to induce liposome fission [38], and the final mitochondrial abscission has been attributed to the Dnm2 GTPase, which is transiently and specifically recruited to the ER- and to Drp1-induced constriction sites, and leads to fission [39]. Nevertheless, recently it has been demonstrated that mouse fibroblasts lacking all three mammalian dynamin proteins (DNM1, DNM2, and DNM3), as well as cells with the single knockdown of DNM2, did not display defects in mitochondrial fission or mitochondrial hyperfusion. In contrast, even a partial reduction of Drp1 caused a robust hyperfusion phenotype, suggesting that mitochondrial fission clearly depends on Drp1 [40].

Furthermore, over the last decade, researchers have focused their attention on an emerging topic, regulating mitochondrial dynamics. Indeed, studies on the fission mechanism demonstrated that Drp1 can undergo different post-translational modifications, such as phosphorylation [41,42], ubiquitination [43,44], SUMOylation [45], O-GlcNAcylation [46], and Nitrosylation [47].

### 2.2. Mitochondrial Fusion

Mitochondrial fusion is a “collision” event, which results in the physical union of two neighboring organelles. At the site of the collision, the fusion process occurs in two steps: Fusion of the outer membrane followed by fusion of the inner membrane. The outcome of this merging process is the transfer of information through the exchange of mtDNA, proteins, lipids, and metabolites [48,49,50]. However, the fusion of two organelles can also occur transiently, through the so-called “kiss-and-run” encounters, during which mitochondria exchange their content at a minor extent, but at the same time, they preserve the original morphology, to sustain their mobility [51].

The fusion machinery consists of three dynamin-related GTPases: Mitofusins 1 and 2 (Mfn1, Mfn2), and optic atrophy 1 protein (OPA1) [52].

Mitofusins, responsible for OMM fusion, contain conserved catalytic GTP-binding domains at the N-terminal and two C-terminal coiled-coil domains (also called heptad-repeat domains, HR1 and HR2) that surround two transmembrane domains I. The two TM domains are separated by a short loop exposing both the N-term and C-term to the cytosol [53]. Mitofusins are directly involved in the docking and fusion of OMM. The docking activity relies on their ability to self-assemble into oligomers within the same membrane or across opposing membranes [54,55]. Two possible models have been proposed for OMM fusion. The first one suggests that the HR2 domain promotes membranes docking throughout the formation of antiparallel coiled-coil mitofusins dimers that bring opposing mitochondrial membranes at ~10 nm from each other; then the HR1 domain, which possesses a conserved amphipathic helix, interacts with the lipid membrane, bringing OMMs in closer proximity and perturbing their lipid bilayer structure [54,56]. In the second one, it is supposed that the GTP binding triggers the Mfns dimers oligomerization and subsequently the GTP hydrolysis leading to a conformational change that pulls the membranes together [56,57,58]. Mfn1 and Mfn2 share approximately 80% sequence similarity in humans, and it has been shown that only when both mitofusins species are deleted the OMM fusion is completely inhibited, clearly demonstrating their redundant nature [59]. Nevertheless, it has been observed that Mfn1-KO induces mitochondrial fragmentation, while Mfn2-KO leads to swollen sphere-shaped mitochondria [59], probably because Mfn1 mediates mitochondrial tethering more efficiently than Mfn2 [55,60], suggesting different roles for these dynamins. Moreover, Mfn1 and Mfn2 expression levels change in different tissues, and Mfn1 expression is higher in more tissues than Mfn2, although the latter is more represented in the heart, brain, and skeletal muscle [60,61]. Interestingly, both a disturbance of the axonal transport, and an altered mitochondrial distribution within neurons after Mfn2 depletion has been highlighted, given the interaction with Miro, an adaptor required for mitochondrial transport across the neuronal axons [62,63]. In addition, similar to mitochondrial division, mitochondrial fusion occurs at ER-mitochondria contact sites as well [64], and Mfn2 was suggested to directly mediate ER-mitochondria tethering in mammals [65,66]. Finally, unlike Mfn1, which acts similarly to other mechanical dynamins, Mfn2 displays high GTP binding and low GTP hydrolysis, exhibiting properties of GTPase signaling as Ras, and a constitutively GTP-bound mutation in Mfn2 did not affect the mitochondrial fusion process [55,67]. Moreover, it has been observed that Mfn2 has an N-terminal Ras-binding domain that is lacking in Mfn1 [68]. Altogether, these observations underline the differences between the two mitofusins and bring out a primary role in mitochondrial tethering for Mfn1 and a regulatory role, closer to the Ras-like GTPases, for Mfn2. The idea that Mfn2 can be a multifunctional protein with roles beyond fusion is supported by several reports describing the involvement of Mfn2 in different signaling pathways, such as mitochondrial bioenergetics, apoptosis, autophagy, and cell cycle progression [69,70,71,72,73,74].

Despite the central role of mitofusins, Misato (MSTO1) has been reported to be involved in the OMM fusion process, since its depletion was found to cause mitochondrial fragmentation [75]. MSTO1 is a soluble cytoplasmic protein that moves to the outer face of the MOM, where it interacts with mitochondrial fusion proteins. It is believed that MSTO1 can support mitochondrial fusion through enhancing or initiating the MOM fusion [76,77]. In addition, mitoPLD, a member of the phospholipase D family, has been proposed to attend the fusion process. MitoPLD is bound to the OM, where it converts cardiolipin to phosphatidic acid, whose negatively charged lipid head group causes negative curvature of lipid bilayers allowing the recruitment of adaptor proteins, required for mitochondrial fusion [78].

Anyway, the key player of inner mitochondrial membrane fusion in mammals is the dynamin-like GTPase OPA1. Similarly, to other dynamins, it contains three highly conserved regions exposed to the inter-membrane space (IMS): The GTP-binding domain, the GTP-effector domain, and the middle domain. In addition, the N-term region, includes a mitochondria targeting sequence followed by a transmembrane helix, required for the IMM anchoring [79,80]. In humans, OPA1 is present in eight different isoforms, that are ubiquitously expressed, although differently expressed in diverse tissues [81]. The OPA1 mRNA variants derive from alternative splicing, and the resulting precursors are targeted to mitochondria via their leader sequence. Once imported into mitochondria, the mitochondrial processing peptidase cleaves the mitochondria targeting sequence, producing the long isoforms of the GTPase (L-OPA1), which is anchored to the IMM [82,83]. L-OPA1 isoforms may be further proteolytically processed at the N-terminus, generating the short forms (S-OPA1) soluble in the IMS, by the action of two inter-membrane enzymes: The mitochondrial metalloendopeptidase OMA1 and the ATP-dependent zinc metalloprotease YME1L [84,85,86]. Unlike YME1L, which is constitutively active, OMA1-dependent cleavage of OPA1 occurs only in response to cellular insults, such as mitochondrial membrane depolarization [87,88]. These proteases regulate mitochondrial morphology fulfilling opposite mechanisms: YME1L-dependent OPA1 processing leads to tubular mitochondrial morphology, while OMA1-dependent OPA1 processing induces mitochondrial fragmentation [89,90]. Nevertheless, what is the role of these two OPA1 versions in the fusion process? Initially, it has been reported that the presence of both L- and S-OPA1 is required for mitochondrial fusion, since L- or S-OPA1 alone is unable to complete the fusion process [86]. Inversely, other works describe only L-OPA1 as fusion competent [89,91,92]. In vitro studies showed that membrane-anchored L-OPA1 binds to cardiolipin (CL) of the opposing membrane to allow tethering and subsequent fusion by GTP hydrolysis, even in the absence of OPA1 in the opposing membrane [93]. These results highlighted two significant features: The presence of OPA1, even only in one of the two opposing mitochondria, can drive the fusion process, while cardiolipin is directly involved in membrane remodeling and dynamics. In fact, the same authors have shown that increased CL content was able to accelerate fusion, while no fusion occurred with no or low CL content [93]. On the other side, the exact role of the short forms of OPA1, generated by the cleavage, is still debated. It has been suggested that L-OPA1 alone is sufficient to ensure fusion, energetics, and cell survival, because OPA1 cleavage leads to the accumulation of nonfunctional S-OPA1, causing mitochondrial fragmentation and cristae disruption, as well as increased susceptibility to apoptotic cell death [94]. Nevertheless, S-OPA1 forms display all the domains typical of other dynamins, also maintaining the GTPase activity, suggesting that they retain functional activities [92]. Contrary to previous reports, it has been described that cells expressing exclusively S-OPA1, failed the fusion process, but successfully maintained cristae structure and energetic activity [92,95], putting forward a hypothesis that S-OPA1 accumulation could have a physiological significance. In addition, recent work showed that under OXPHOS and oxidant conditions, S-OPA1 depletion strikingly increases oxidant-induced cell death, putting in evidence that S-OPA1, and not L-OPA1, is able to counteract oxidative stress extending cell survival [92]. However, besides the energetic and antioxidant competencies, some authors suggested a direct role of S-OPA1 in IMM fusion: In vitro studies revealed that the addition of S-OPA1 to the L-OPA1-containing fusion mixture increased fusion efficiency [93], and robust fusion was observed when S-OPA1 was mixed with CL-rich liposomes [96], similar results have been reached in cultured cells where OPA1 cleavage is required to obtain IM fusion as well [90]. In addition, a very recent study, through the use of an in vitro reconstitution approach, demonstrated that S-OPA1 alone can carry out the membrane tethering, it is unable to complete the merge of lipids in the two bilayers. In contrast, L-OPA1 can tether and hemifuse bilayers, but is insufficient to mediate the final step of fusion, consisting in the pore opening, while the presence of both S-OPA1 and L-OPA1 provides an efficient and fast pore opening, allowing a complete fusion process [97].

Another protein involved in mitochondrial fusion is F-box and leucine-rich repeat 4 (FBXL4). This protein, localized in the mitochondrial inter-membrane space, comprises a leucine-rich repeat domain, required for protein-protein interactions and through which it forms quaternary protein complexes [98]. A role in the mitochondrial fusion process for FBXL4 has been supposed, because defects in this protein resulted in mitochondria fragmentation [98,99,100], while overexpression of wild-type FBXL4 promoted mitochondrial hyperfusion [101].

Post-translational modifications of mitochondrial fusion proteins are less documented. Besides the OPA1 protein regulation by proteolytic cleavage above described [84,85,86], only a few modifications have been associated with Mfn1 and Mfn2. The activity and stability of Mfn1 are regulated by ubiquitination and acetylation [102,103], while Mfn2 can undergo ubiquitination [104,105].

## 3. Mitochondrial Dynamics Related Disorders

The central role of mitochondria in cellular health and survival explains why so many regulatory mechanisms are dedicated to the maintenance of mitochondrial homeostasis. Among the processes aimed at the defense of mitochondrial functions, mitochondrial dynamics are at the forefront, because fusion and fission machinery, through the control of mitochondrial morphology, take part in several cellular processes, including energy metabolism, apoptotic cell death, autophagy, and immune signaling [5,106,107]. For this reason, defects in mitochondrial dynamics have been associated with different human disorders, such as cancer, metabolic syndromes, and neurodegenerative disorders. In this section, we will focus our attention on fission and fusion defects related to MD.

### 3.1. Fission Related Mitochondrial Diseases

#### 3.1.1. Drp1

**Figure 2:**
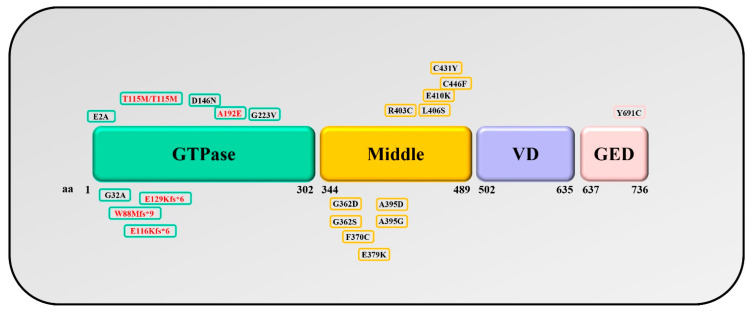
**Schematic representation of Drp1 domains**. Distribution of the mutations reported up to date. In red, homozygous or compound heterozygous variants; in black, dominant-negative variants. It should be noted that most of the dominant variants are located in the middle domain. GED, GTPase effector domain; VD: variable domain.

Drp1 defects are implicated in complex neurological disorders known as “Encephalopathy due to defective mitochondrial and peroxisomal fission 1” (EMPF1, OMIM #603850). Affected patients display variable phenotypes ranging from severe hypotonia, to psychomotor delay, with or without seizures. The first pathogenic variant in the DNM1L gene was identified in a patient with a severe clinical course, characterized by microcephaly, optic atrophy, hypoplasia, persistent lactic acidemia, and neonatal death [108]. Following this first description, no additional patients were reported for about 10 years, but more recently, several studies have described an increasing number of patients, expanding the range of phenotypes. DNM1L mutations have been found in the GTPase, middle, and GED domains (Figure 2).

All the variants affecting the middle domain are reported as de novo heterozygous mutations, and are supposed to act as a dominant-negative, impairing the protein oligomerization. These variants give rise to neonatal or infantile-onset encephalopathy manifesting with hypotonia, weakness, psychomotor delay, drug-resistant seizures, microcephaly, and growth failure. Some patients presented poor feeding and respiratory insufficiency, probably arising from hypotonia and weakness, while optic atrophy has been reported only occasionally. Neuroimaging frequently showed delayed myelination and cerebral atrophy. Biochemical investigations revealed reduced activity of mitochondrial respiratory chain in muscle and elevated serum lactate in less than half of the described patients. In all cultured fibroblasts examined, mitochondria, and in some cases, peroxisomes appear elongated and hyperfused [108,109,110,111,112,113,114,115,116,117,118,119,120,121,122,123]. Although muscle biopsies of patients have been poorly investigated from a histological point of view, recently, our group reported five patients with mutations in *DNM1L* displaying histological muscle anomalies consisting of abnormal distribution of mitochondria in muscle fibers not associated with mitochondrial DNA instability, and these abnormalities seem to be specific for the DMN1L-related epileptic encephalopathy [119]. Besides the common clinical signs, a Drp1 middle domain defect has also been associated with severe infantile parkinsonism with prominent hypokinetic-rigid syndrome, high amplitude rest tremor, and oculogyric crises without extra neurological involvement [114]. In addition, a dilated left ventricle with decreased systolic function was described in a patient, and postmortem investigations confirmed the cardiac involvement given the presence of myocytes with enlarged mitochondria, separation of myofibrils, interstitial fibrosis, and ventricular hypertrophy and dilation [121].

Concerning the GTPase domain, other authors have found both monoallelic and biallelic pathogenic variants. Heterozygous mutations have been described in three French families, supposed to act with a restricted dominant-negative effect [124]; patients displayed a slow decrease in visual activity, dyschromatopsia, central scotoma, and optic atrophy with onset in the first to third decade of life, while no other neurological signs were reported [124]. Contrariwise, three additional heterozygous mutations have been associated with severe neurological phenotype showing encephalopathy, drug-resistant seizures, hypotonia, psychomotor developmental delay, and sensory axonal neuropathy [117,119,125], and only in one case, the neurological signs were accompanied by ocular involvement [117]. Moreover, heterozygous compound and homozygous missense variants were reported in five children from three families and were shown to result in loss of function. Affected patients presented clinical symptoms at birth or during infancy, displaying delayed psychomotor development, weakness, hypotonia, and oculomotor apraxia. Serum lactate was increased in three of the five patients, while an isolated partial complex IV deficiency was documented in fibroblasts from only one patient. Moreover, death during infancy was reported in two affected children [126,127,128].

Recently, for the first time, a patient has been described with a de novo heterozygous variant in the GED domain of Drp1. The patient is a 27-year-old girl with a history of neonatal hypotonia progressed to spasticity, developmental delays, and static encephalopathy. Functional studies in Drosophila suggest a dominant-negative mechanism effect of this variant, due to the mutation failed to rescue Drosophila drp1 lethality and caused an altered peroxisomal phenotype [129].

#### 3.1.2. Mff

Biallelic pathogenic variants in the MFF gene are recognized as the cause of the so-called “Encephalopathy due to defective mitochondrial and peroxisomal fission 2” (EMPF2; MIM#617086). Seven patients from five families have been described, and all of them presented with severe neurological signs, including delayed psychomotor development, severe hypotonia, external ophthalmoplegia, feeding, and seizures. Neuroimaging revealed basal ganglia signal abnormalities. Cultured fibroblasts from patients displayed tubular, elongated, and hyperfused mitochondria and peroxisomes, indicating organelle fission defects [130,131,132,133].

#### 3.1.3. MIDs

In 2018, for the first time, a homozygous nonsense mutation in the gene encoding MID49 protein was reported in a patient with an isolated mitochondrial myopathy. Although mitochondrial fission defects are mostly associated with the impairment of the central nervous system, this patient was affected by an isolated myopathy. The diagnostic investigations revealed increased creatine kinase and both histological and biochemical alterations of muscle biopsy, including the presence of numerous ragged red fibers (RFF), COX negative fibers, and reduced activities of the mitochondrial respiratory chain complexes I and IV. Moreover, patient fibroblasts displayed elongated mitochondria and significantly increased fusion events, suggesting a defect in mitochondrial dynamics. The MID49 defect was confirmed by rescue experiments, showing that wild-type MID49 was able to recover mitochondrial morphology and dynamics, confirming that the identified mutation was the cause of the abnormal mitochondrial hyperfusion phenotype [134].

### 3.2. Diseases Related to Mitochondrial Fusion Abnormalities

#### 3.2.1. OPA1

OPA1 defects can arise from both monoallelic and biallelic pathogenic variants. Heterozygous mutations have been associated with the dominant optic atrophy (DOA) (OPA1; MIM# 165500), which is the most frequent form of hereditary optic neuropathy. The main consequence of the OPA1-related DOA is the degeneration of retinal ganglion cells, the axons of which form the optic nerve, resulting in optic atrophy, which is frequently bilateral and symmetric. The disease usually manifests in childhood, but clinical signs can also appear from the third decade of life. In most reported cases, visual acuity worsens slowly and vision loss, is progressive and irreversible. Ophthalmologic impairments can also include visual field and color vision defects [135]. Up to 20% of patients develop a syndromic DOA phenotype, named DOA “plus” (DOA+; MIM# 125250) syndrome, which manifests with additional extra-ocular features, such as sensorineural hearing impairment, proximal myopathy, exercise intolerance, ataxia, peripheral neuropathy, and ophthalmoplegia [136]. Histological analysis of muscle biopsies can reveal RRF, COX negative fibers, and subsarcolemmal abnormal mitochondria proliferation; in addition, mtDNA instability has been identified in DOA-plus patients [137], even if mtDNA deletions are also present in patients with isolated DOA [138]. Altered mitochondrial morphology, oxidative stress, and dysfunctional oxidative phosphorylation are documented in cells derived from OPA1 patients [139]. To date, more than 400 monoallelic variants have been reported, most of which are single base-pair substitutions, followed by deletions, and insertions [140]. Mutations have been found along the coding sequence of the gene, but the GTPase and the C-terminal coiled-coil domains seem to be mutational hot spots, resulting in dominant-negative and haploinsufficiency phenotypes, respectively [141].

Biallelic pathogenic variants in the OPA1 gene are associated with Behr syndrome (MIM# 210000). The first patient was reported in 2001 harboring two heterozygous missense mutations in exon 8 of the OPA1 gene, and the clinical examinations showed that the compound heterozygous patient was the most severely affected family member, with serious vision defects and pallor of the optic discs, but no neurological signs were documented [142]. Over the next nearly ten years, no biallelic mutations were described in OPA1 patients, but in 2010 two distinct mutations in OPA1 were found in two siblings showing optic atrophy and deafness without neurological symptoms, and in the same report, compound heterozygous mutations were identified in two adult siblings presenting with optic atrophy, ataxia, myopathy, neuropathy, and spasticity. However, the absence of parental DNA samples did not allow to segregate the compound heterozygosity for none of these patients [136]. Since 2011 14 patients from 13 families have been reported to harbor compound heterozygous or homozygous mutations. Most of the cases showed an early-onset OPA1-related syndromic phenotype, different from those previously described and consisting of neurological signs compatible with the Behr syndrome. These patients were identified during infancy or early childhood with optic neuropathy and spinocerebellar degeneration, pyramidal signs, peripheral neuropathy, gastrointestinal dysmotility, and delayed motor development. Neuroimaging typically revealed cerebellar vermian atrophy, and periventricular white matter alterations, but also a Leigh-like brain MRI, with basal ganglia hyperintensities, and pathological lactate accumulation was detected. When examined, fibroblasts from affected individuals displayed fragmented mitochondrial network, while histological investigations showed increased RRF in one patient and diminished COX reactivity in two cases. Biochemical findings in muscle biopsies revealed a decreased complex IV activity only in one patient. The parents of the affected individuals presented with variable phenotypes ranging from optic atrophy to asymptomatic state, suggesting a reduced penetrance [143,144,145,146,147,148].

However, biallelic mutations in OPA1 have also been associated with other severe neurological conditions. Two sisters, harboring homozygous mutations, have been reported with a very serious clinical picture: Developmental delay, muscle weakness and wasting, poor feeding, failure to thrive, hypertrophic cardiomyopathy, hypertonia, optic atrophy, sensorineural hearing impairment, and lactic acidemia. Both patients died following apneic episodes. Biochemical examinations on muscle biopsies revealed reduced electron transport chain activities, and mtDNA depletion were also identified in skeletal muscle [149]. Recently a twelve-year-old girl was described harboring biallelic OPA1 mutations, presenting with a complex neurological disorder. The patient exhibited early-onset optic atrophy, progressive gait ataxia, dysarthria, tremor, and learning impairment. In addition, at the age of 12 years, a metabolic stroke occurred [150], expanding the clinical manifestations of OPA1-related disorders.

Finally, in 2014, for the first time, two deep intronic mutations in intron 4b of the OPA1 gene in a total of four families were detected. The authors showed that these intronic variants were the cause of optic atrophy observed in the patients. They also demonstrated that certain OPA1 sequence alterations can act as intralocus disease-modifiers, and occur in a compound-heterozygous state, such modifiers can cause optic atrophy plus disease, following the model of a combined mutational effect [151].

#### 3.2.2. YME1L1

Homozygous YME1L1 missense mutation has been identified in four siblings with an infantile-onset mitochondriopathy. The affected children presented with intellectual disability, motor developmental delay, expressive speech delay, hearing impairment, and optic nerve atrophy associated with visual impairment. Inconsistent features were microcephaly or macrocephaly, ataxia, hyperkinesia, athetotic and stereotypic movements. Neuroimaging showed leukoencephalopathy in all four patients and signs of brain atrophy in two of them. Lactate levels were elevated in blood and/or cerebrospinal fluid of three patients. Histological investigations on muscle biopsy from one patient revealed mitochondria with altered cristae morphology, while patient fibroblasts displayed shortened and fragmented mitochondrial networks consistent with the fusion defect [152].

#### 3.2.3. MFN2

MFN2 dysfunctions can originate from both monoallelic and biallelic pathogenic variants. Monoallelic mutations are associated with the Charcot-Marie-Tooth neuropathy type 2A (CMT2A; MIM# 609260), the optic atrophy, and the hereditary motor and sensory neuropathy VIA (HMSN VIA; MIM# 601152). CMT2A is an axonal non-demyelinating peripheral neuropathy, manifesting in the first or second decade of life, with an estimated incidence of 1–3:100,000 [141,153], and is considered one of the most four frequent neuropathies, and the most frequent CMT2 (axonal neuropathy) [154]. The clinical features of the classical form of CMT2A include muscle weakness and atrophy, which predominantly affect the distal lower limbs, but progressively can also involve the upper extremities. In the early stages of the disease, motor symptoms can include physical weakness, walking difficulty, and foot deformities (pes cavus). Skeletal deformities, in particular severe scoliosis, may occur. Besides the motor symptoms, patients can complain of sensory loss in the feet and areflexia. Additional clinical signs, often observed in early-onset cases, are optic atrophy, sensorineural hearing impairment, hoarse voice, and contractures. The disease has a progressive course, even if life span is typically not reduced, and generally, early-onset patients show a more severe clinical course than those with late-onset. Nerve biopsies of affected individuals usually show loss of large, myelinated fibers, mitochondrial abnormalities, but no myelin alterations. Uncommon presentations are related to CNS involvement, such as pyramidal and white-matter alterations or encephalopathies; in fact, in a minority of patients’ neuroimaging revealed periventricular and subcortical white matter lesions [62,141].

MFN2 monoallelic mutations have also been clinically linked to optic atrophy disease, and several cases have been reported in the literature. In all the patients described optic atrophy typically started during early childhood, causing vision impairment, central scotomas, and color vision deficiency [141,155,156,157,158,159,160]. In one large family, the optic atrophy resembled the DOA “plus” phenotype related to OPA1 mutations, showing neuropathy and mitochondrial myopathy in adult life. Skeletal muscle tissue displayed COX-deficient fibers, RFF, and multiple mtDNA deletions, while in patient fibroblasts were observed a reduced mitochondrial fusion and a lower capacity to repair stress-induced mitochondrial DNA lesions compared to control cells. [156]. Very recently, a patient with a chronic onset optic neuropathy with normal visual acuity was reported, who had no impaired color vision, but the severe concentric narrowing of the visual field [160].

Autosomal recessive pathogenic variants in MFN2 gene have been identified in 29 patients, many of which presented with severe axonal neuropathy. Age of onset was generally early childhood for the majority of the reported cases, even if recently a patient with an adult-onset has been described. Foot drop is the most frequent initial symptom. Most affected individuals showed moderate or severe neuropathy. Proximal weakness with moderate to severe distal weakness was described. Skeletal deformities, such as kyphosis, scoliosis, and pes cavus, were commonly reported, whereas vision abnormalities were less frequent. Other clinical findings included hearing loss, lipodystrophy, vocal cord palsy, tongue hypertrophy, and facial and respiratory muscle weakness. Heterozygous carrier parents showed no or minimal symptoms [157,161,162,163,164,165,166,167,168,169,170,171,172].

The majority of the identified MFN2 mutations fall in the GTPase domain and in the downstream region before the HR1 domain [173]. The arginine at position 94, in the P-loop G1 subdomain, seems to be the residue most prone to mutations, probably because this position is located within a hotspot region for mutation [161]. Moreover, most of the pathogenic variants are reported as missense mutations, which can lead to gain of function or loss of function, causing the protein accumulation in mitochondria or mitochondrial fusion impairment, respectively [161,164].

#### 3.2.4. MSTO1

MSTO1 pathogenic variants have been identified in 27 patients from 19 families and have been associated with mitochondrial myopathy and ataxia (MMYAT; MIM# 617675). All patients carried heterozygous compound or homozygous mutations, and in only one family, the mutation was reported to be inherited as an autosomal dominant trait. The age of onset can range from early childhood to adulthood. The common clinical features documented were myopathy, cerebellar atrophy, and ataxia [76,77,174,175,176,177]. Myopathy manifested with muscle weakness, motor developmental delay, mildly or severely elevated CPK, myopathic pattern in EMG, and myopathic and dystrophic alterations on muscle biopsy. Other clinical signs were growth failure, pigmentary retinopathy, hypotonia, skeletal deformities, and cognitive impairment. In one family, some of the affected individuals showed psychiatric and behavioral manifestations as well, such as anxiety, autistic features, depression, and schizophrenia, and endocrinopathies, including hyperthyroidism, hyperprolactinemia, and vitamin D deficiency [76]. Neuroimaging revealed stable or progressive cerebellar atrophy, and in some cases, the upper motor neuron signs and the substantial clinical progression over time, suggested a more complex and progressive neurological phenotype [176]. When analyzed, the patient’s fibroblasts displayed fragmented mitochondria and mtDNA depletion. Moreover, patient fibroblasts also showed enlarged lysosomal vacuoles and a reduced number of nucleoids, but larger in size compared to control lines [176].

#### 3.2.5. FBXL4

FBXL4 biallelic pathogenic variants have been associated with encephalomyopathic mtDNA depletion syndrome 13 (MTDPS13; OMIM # 615471), a multisystem disease that mainly affects the central nervous system, heart, and liver, occurring with an estimated prevalence of 1:100,000–400,000. The onset is typically early; patients show clinical signs in the neonatal period. One hundred one cases of FBXL4 deficiency have been reported so far. MTDPS13 typically manifests with failure to thrive, neurodevelopmental delay, encephalopathy, cerebral atrophy, hypotonia, and persistent lactic acidosis. Usually, affected individuals have microcephaly and hyperammonemia. However, given the multisystemic nature of the disease, clinical signs can involve any apparatus. Neurologic manifestations can include seizures, ataxia, sensorineural hearing impairment, and movement disorders. Ocular defects observed are strabismus, nystagmus, and optic atrophy. Cardiac involvement has been described in some patients with cardiomyopathy, congenital heart diseases, and arrhythmias. Occasionally, also gastrointestinal, genitourinary, and immunologic manifestations have been reported, as well. Neuroimaging generally reveals hypomyelination, cerebral atrophy, basal ganglia abnormalities, dilated ventricles, corpus callosum thinning, cerebellar atrophy, and brain stem abnormalities. Skeletal muscle commonly shows mitochondrial dysfunctions with combined deficiencies of multiple OXPHOS complexes and mtDNA depletion. Patient’s fibroblasts typically display fragmented mitochondria and combined deficiencies of multiple OXPHOS complexes [98,99,100,101,130,178,179,180,181,182,183,184,185,186,187,188,189].

## 4. Therapeutic Approaches

The diagnosis and treatment of mitochondrial diseases are challenging because of the high genetic and clinical heterogeneity of these disorders. However, in the last ten years, the development of new sequencing technologies has allowed the resolution of many undiagnosed cases. Moreover, the identification of new disease genes has given the possibility to uncover different pathways involved in the pathogenic process and potentially targetable, paving the way for therapeutic approaches. To date, an effective cure is not yet available for mitochondrial patients. For very severe MDs, management involves predominantly supportive care and the early treatment of organ-specific complications, while for patients with less-severe MDs, the primary aim is to reduce morbidity and mortality.

In the last years, the use of small molecules, both in preclinical studies and in several clinical trials, has resulted in a good strategy for the MDs treatment, and several biotech companies are dedicated to the development of new drugs to counteract mitochondrial pathologies. This interest is also motivated by the fact that mitochondria may represent the therapeutic target for other pathologic conditions, such as Parkinson’s disease, Alzheimer’s disease, amyotrophic lateral sclerosis, Huntingdon’s disease, Friedrich ataxia, and metabolic syndromes, including obesity and diabetes [190], making the development of mitochondrial modifiers more attractive.

A promising therapeutic strategy is gene therapy, which consists of the complementation of the mutant gene by expressing the wild-type allele, through the use of viral vectors, such as adeno-associated viral vectors (AAVs), characterized by a safety profile, and tissue-specific serotypes [191]. However, this approach shows some limitations related to the low cloning capacity and the resulting difficulty in reaching therapeutic expression levels in affected tissues, making it applicable to diseases involving small genes and a single organ or site. Actually, AAVs-based gene therapy is carried out in mouse models of ADOA, Mitochondrial Neurogastrointestinal Encephalomyopathy, Ethylmalonic Encephalopathy and Leigh syndrome [192,193,194,195], and several AAV-based clinical trials are registered for LHON disease [196]. Table 1 summarizes all the therapeutic approaches used up to date.

### 4.1. Correcting DNM1L-Related Defects

To date, no therapeutic options are available for patients with *DNM1L* defects. As discussed above, affected individuals present with severe encephalopathy, and a common clinical sign is a generalized status epilepticus, usually refractory to most of the anticonvulsants.

Recently, promising results from a preclinical study that demonstrated the ability of bezafibrate to improve mitochondrial fission and function in *DNM1L*-deficient patient cells, were reported [197]. Bezafibrate is a small molecule agonist of the peroxisome proliferator-activated receptor alpha (PPARα), a ligand-activated transcription factor that, upon activation, increases the expression of several target genes, including nuclear-encoded respiratory chain genes [202]. Douiev in 2020 demonstrated that bezafibrate treatment is effective in normalizing growth, ATP production, and oxygen consumption in patient’s fibroblasts harboring the p.G362S variant in the DNM1L gene. Moreover, the treatment showed a beneficial effect also on mitochondrial morphology, decreasing the abnormal elongated phenotype. The positive effects of bezafibrate on energy metabolism can be attributed to its ability both to increase fatty acid utilization and to stimulate mitochondrial biogenesis, whereas its effects on mitochondrial morphology suggests that it could target other mitochondrial processes, such as mitochondrial dynamics and quality control. However, we must consider the possible side negative effects, in fact, the treatment with bezafibrate seems to increase ROS production, probably due to increased respiratory chain activity and mitochondrial content [197].

### 4.2. OPA1 Related Therapies

In the past, the treatment of blindness in patients with mitochondrial optic neuropathies was based on the administration of various vitamins, such as vitamins E and C, and other compounds, including alpha-lipoic acid, l-carnitine, creatine, l-arginine, and cysteine, but these therapeutic attempts have led to poor results [203]. Common features observed in fibroblasts from OPA1 patients are the reduced activity of respiratory complex I, and increased level of ROS [204,205]. These peculiarities suggested the administration of ubiquinone analogs and antioxidants, extensively used in patients with mitochondrial disorders. Among the ubiquinone analogs, coenzyme Q10 (CoQ10) was used to bypass the affected complex I, but resulted in no evidence for a significant benefit [198]. Promising results have been achieved with idebenone, a short-chain analog of ubiquinone with antioxidant competence: It was reported that 74 of 87 DOA patients displayed increased visual acuity after at least seven months of idebenone administration [199]. A recent work used a “drug repositioning” approach to identify FDA-approved molecules, which are able to rescue the mitochondrial dysfunctions induced by OPA1 mutations. The combined use of yeast, mouse embryonic fibroblasts (MEFs), and human model has allowed an accurate selection of the screened molecules: Twenty-six drugs were effective in the rescue of the oxidative growth phenotype in yeast strains carrying mgm1 mutations; six of them, acting on mtDNA instability, have been used to treat Opa1 deleted MEFs, and some of them showed a positive effect on energetic metabolism and/or mitochondrial morphology; the final validation on patient’s fibroblasts has led to the selection of the tolfenamic acid as the most promising compound to be used in a clinical trial for patients with OPA1 mutations [200].

A therapeutic approach with high potentialities for DOA patients is represented by gene therapy, since DOA is a monogenic disease, and the eye is a good candidate for this type of therapy as target genes can be delivered directly to the retinal ganglion cells via intravitreal injection. Several AAV-based clinical trials are registered for LHON disease, and the first results show a potential treatment effect with progressive visual field improvement [196]. These encouraging results give hope that AAV-based gene therapy can also be designed for OPA1-related DOA. The first evidence of the gene therapy feasibility comes from a recent work, showing the efficiency of the AAV2 construction to transduce and maintain both long and short OPA1 isoforms expression in the mouse retinal ganglion cells, preventing their degeneration. In spite of the positive effects on the electrophysiological measurements specific of retinal ganglion cell activity, visual acuity slightly improved in Opa1+/− treated mice, suggesting that OPA1 gene therapy may be sufficient to prevent retinal ganglion cell degeneration, but the number of transfected cells was insufficient to achieve the improvement of the electric activity along the optic path [192]. More challenging appears the application of gene therapy in case of missense mutations, associated with the DOA “plus” or Behr syndrome phenotypes. In these cases, the mutations act in a dominant-negative mode, and the increase of wild-type OPA1 levels may not be sufficient to ameliorate the clinical phenotype. It will be necessary to develop a mouse model carrying missense mutations, to test the efficacy of OPA1 gene therapy in the presence of a dominant-negative allele, and even more difficult appears the delivery to multiple tissue targets.

### 4.3. Therapeutic Perspectives for MFN2-Related Disorders

The cellular and molecular mechanisms by which MFN2 mutations lead to neuronal degeneration are not yet clear, and no effective disease-modifying treatment is available for patients. Affected individuals are undergoing supportive care, including exercise, physical, and occupational therapy and orthotics designed to address foot drop symptoms and to stabilize gait [206,207,208]. Although MFN2 is ubiquitously expressed, and its functioning is essential for many tissues [59,209,210,211], MFN2 mutations preferentially affect the nervous system. The causes of this neuronal specificity can be attributed to both the low level of MFN1 expression in the nervous system and to the importance of energy metabolism at the axon level, strictly dependent on adequate mitochondrial transport and localization [63,212]. However, in vitro studies showed that increasing MFN1 expression is sufficient to counteract mitochondrial fusion and transport defects caused by MFN2 loss [63,213]. Recently, the ability of MFN1 to compensate for the MFN2 loss has also been demonstrated in vivo. A transgenic mouse generated with the MFN2 point mutation MFN2^R94Q^, presents neurologic features seen in CMT2A patients, such as severe early-onset sensorimotor deficits, vision loss, and axonal degeneration, and it was shown that increased expression of MFN1 in the nervous system rescued the MFN2 mutant phenotypes, highlighting the importance of MFN isoforms balance and providing a potential therapeutic strategy for this disease [214].

Promising therapeutic approaches come from mitofusin agonists, which miming the MFN2 peptide–peptide interface, can allosterically activate the protein and stimulate the mitochondrial fusion [201]. It has been reported that the treatment of cultured MFN2 mutated neurons with these molecules, restored mitochondrial dysmotility and fragmentation. In addition, administration of the mitofusin agonists to MFN2^T105M^ transgenic mice via sciatic nerve injection was able to normalize the axonal mitochondrial trafficking within sciatic nerves, pointing out the therapeutic potential of these small molecules [201].

## 5. Concluding Remarks

Mitochondria constitute an interconnected community, governed by the constant and opposite processes of fission and fusion, their shape, size, and positioning, and are critical for the metabolic needs of the cell, cellular homeostasis, and function. Moreover, the maintenance of the mitochondrial architecture is required for the mitochondrial quality control mechanisms, such as mitophagy.

## Figures and Tables

**Table 1:** Therapeutic approaches.

Small Molecules			
Genes Related-Disease	Treatment	Outcome	Reference
DNM1L	bezafibrate	Improvement of growth, adenosine triphosphate (ATP) production, and oxygen consumption in patient’s fibroblasts harboring the p.G362S variant in the DNM1L gene	[197]
OPA1	coenzyme Q10	No evidence for a significant benefit in dominant optic atrophy (DOA) patients	[198]
	idebenone	In 74 of 87 DOA treated patients, an increased visual acuity was observed after at least 7 months of administration	[199]
	tolfenamic acid	Positive effects on mtDNA instability energetic metabolism and/or mitochondrial morphology in the yeast model, Opa1 deleted MEFs and patient’ fibroblasts	[200]
MFN2	mitofusin agonists	Recovery of mitochondrial dysmotility and fragmentation in cultured MFN2 mutated neurons and normalization of the axonal mitochondrial trafficking within sciatic nerves in MFN2T105M transgenic mice	[201]
Gene therapy			
OPA1	AAV2 serotype 2 injection	Positive effects on the electrophysiological measurements specific of retinal ganglion cell activity in Opa1+/− treated mice, but the slow improvement of visual acuity	[192]
MFN2	Thy1.2-MFN2R94Q transgenic mice and Thy1.2-MFN2R94Q:Prp-MFN1 double-transgenic animals	Restoration of MFN1:MFN2 balance by augmenting levels of MFN1 in the nervous system showed near complete rescue of ocular, neuromuscular, and histologic phenotypes	[201]

## Data Availability

Not applicable.
